# The genome sequence of the 6-spot burnet, *Zygaena filipendulae* (Linnaeus, 1758)

**DOI:** 10.12688/wellcomeopenres.17924.1

**Published:** 2022-08-01

**Authors:** Douglas Boyes, Liam M. Crowley, University of Oxford and Wytham Woods Genome Acquisition Lab, Darwin Tree of Life Barcoding collective, Wellcome Sanger Institute Tree of Life programme, Wellcome Sanger Institute Scientific Operations: DNA Pipelines collective, Tree of Life Core Informatics collective, Chelsea Skojec, David Plotkin, Akito Y. Kawahara, Darwin Tree of Life Consortium

**Affiliations:** 1 UK Centre for Ecology and Hydrology, Wallingford, Oxfordshire, UK; 2 Department of Zoology, University of Oxford, Oxford, UK; 3 Kawahara Lab, University of Florida, Gainesville, USA; 4 McGuire center for Lepidoptera and Biodiversity, Florida Museum of Natural History, Gainsville, USA

**Keywords:** Zygaena filipendulae, 6-spot burnet, genome sequence, chromosomal, Arthropoda

## Abstract

We present a genome assembly from an individual female *Zygaena filipendulae* (6-spot burnet; Arthropoda; Insecta; Lepidoptera; Zygaenidae). The genome sequence is 365.9 megabases in span. The majority of the assembly (99.99%) is scaffolded into 31 chromosomal pseudomolecules, with the W and Z sex chromosomes assembled. The complete mitochondrial genome was also assembled and is 15.6 kilobases in length. Gene annotation of this assembly on Ensembl has identified 12,493 protein coding genes.

## Species taxonomy

Eukaryota; Metazoa; Ecdysozoa; Arthropoda; Hexapoda; Insecta; Pterygota; Neoptera; Endopterygota; Lepidoptera; Glossata; Ditrysia; Zygaenoidea; Zygaenidae; Zygaeninae; Zygaena; *Zygaena filipendula*e (Linnaeus, 1758) (NCBI:txid287375).

## Background

The six-spot burnet moth, *Zygaena filipendulae* (Linnaeus, 1758) is an aposematic, chemically defended, day-flying moth in the family *Zygaenidae* with a distribution that ranges across Europe. There are 98 described species of burnet moths in *Zygaena* ( Hofmann & Gerald Tremewan, 2005). Some *Zygaena* species have become model organisms to study the evolution of chemical defence compounds ( Zagrobelny *et al.,* 2019). Forewings of *Z. filipendulae* are black and distinctively marked with six red spots. This species can biosynthesize cyanogenic glucosides *de novo*, or obtain them from *Fabaceae* host plants, storing cyanoglucosides in cuticular cavities and hemolymph, for later use as a defensive secretion ( Franzl *et al*., 1986). The three enzymes involved in the evolution of biosynthesis in *Z. filipendulae* are cytochrome CYP405A2, CYP332A3, and glucosyl transferase UGT33A1 ( Zagrobelny *et al.*, 2019). A genome of *Z. filipendulae* is much needed especially in order to understand the genetics of cyanogenic glucoside biosynthesis.

## Genome sequence report

**Figure 1.**
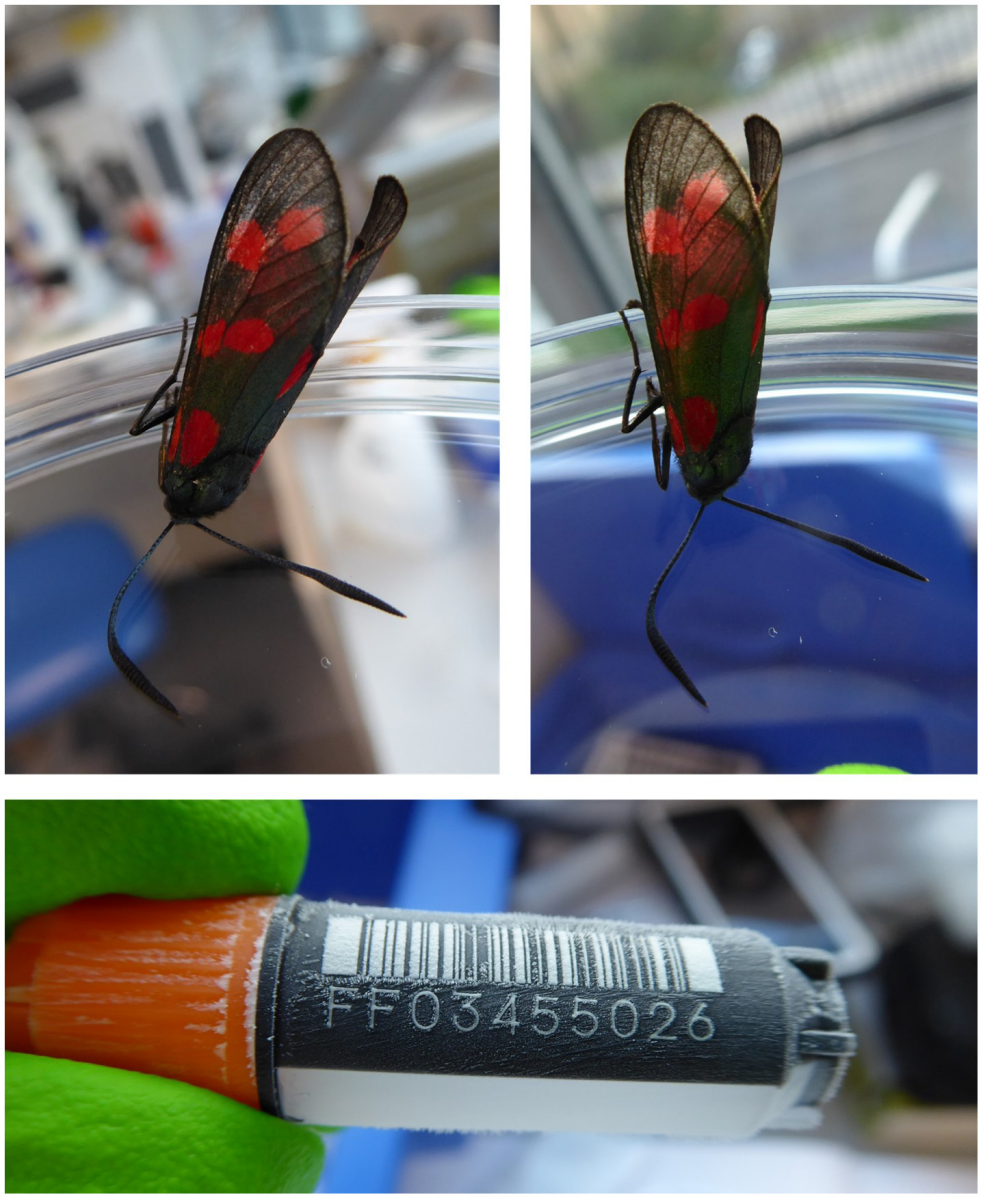
Image of the *Zygaena filipendulae* specimen taken prior to preservation and processing.

The genome was sequenced from a single female *Z. filipendulae* collected from Ant Hills region, Wytham, Berkshire, UK ( Figure 1). A total of 58-fold coverage in Pacific Biosciences single-molecule HiFi long reads and 92-fold coverage in 10X Genomics read clouds were generated. Primary assembly contigs were scaffolded with chromosome conformation Hi-C data. Manual assembly curation corrected 3 missing/misjoins and removed 0 haplotypic duplications, reducing the assembly size by 0.004% and the scaffold number by 8.33% and the scaffold N50 remained the same.

**Figure 2.**
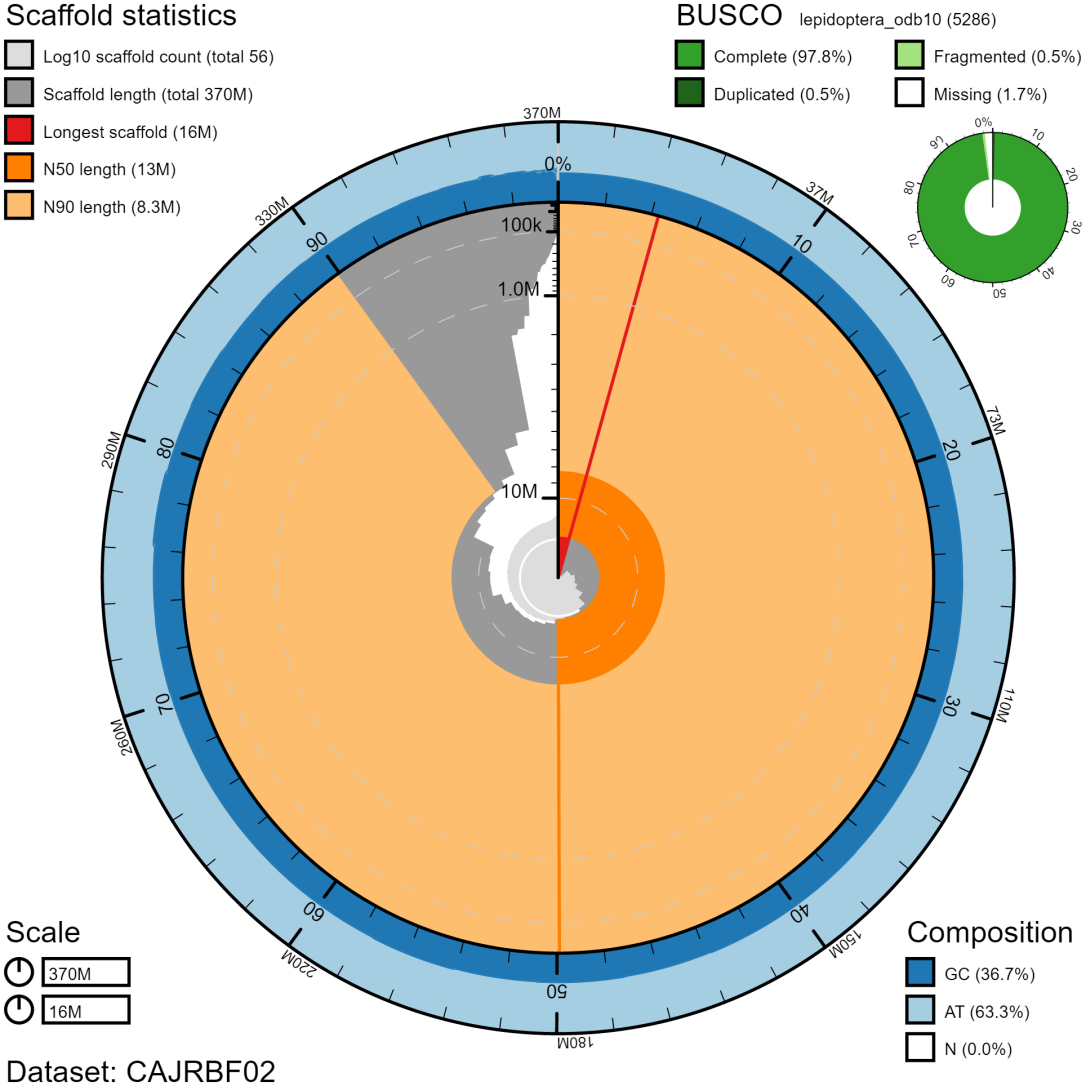
Genome assembly of *Zygaena filipendulae*, ilZygFili1.2: metrics. The BlobToolKit Snailplot shows N50 metrics and BUSCO gene completeness. The main plot is divided into 1,000 size-ordered bins around the circumference with each bin representing 0.1% of the 365,946,273 bp assembly. The distribution of chromosome lengths is shown in dark grey with the plot radius scaled to the longest chromosome present in the assembly (16,101,494 bp, shown in red). Orange and pale-orange arcs show the N50 and N90 chromosome lengths (12,640,274 and 8,250,661 bp), respectively. The pale grey spiral shows the cumulative chromosome count on a log scale with white scale lines showing successive orders of magnitude. The blue and pale-blue area around the outside of the plot shows the distribution of GC, AT and N percentages in the same bins as the inner plot. A summary of complete, fragmented, duplicated and missing BUSCO genes in the lepidoptera_odb10 set is shown in the top right. An interactive version of this figure is available at https://blobtoolkit.genomehubs.org/view/ilZygFili1.2/dataset/CAJRBF02/snail#Filters.

The final assembly has a total length of 365.9 Mb in 55 sequence scaffolds with a scaffold N50 of 12.6 Mb ( Table 1). The majority, 99.99%, of the assembly sequence was assigned to 31 chromosomal-level scaffolds, representing 29 autosomes (numbered by sequence length) and the W and Z sex chromosomes ( Figure 2– Figure 5; Table 2).

The assembly has a BUSCO v5.2.2 ( Manni *et al.*, 2021) completeness of 97.8% (single 97.3%, duplicated 0.5%) using the lepidoptera_odb10 reference set (n=954). While not fully phased, the assembly deposited is of one haplotype. Contigs corresponding to the second haplotype have also been deposited.

**Table 1.** Genome data for *Zygaena filipendulae*, ilZygFili1.2.

*Project accession data*	
Assembly identifier	ilZygFili1.2
Species	*Zygaena filipendulae*
Specimen	ilZygFili1 (genome assembly); ilZygFili2 (Hi-C); ilZygFili3 (RNA-Seq)
NCBI taxonomy ID	287375
BioProject	PRJEB44832
BioSample ID	SAMEA7519846
Isolate information	Female, whole organism (ilZygFili1); head/thorax tissue (ilZygFili2); abdomen tissue (ilZygFili3)
*Raw data accessions*	
PacificBiosciences SEQUEL II	ERR6436369
10X Genomics Illumina	ERR6054694-ERR6054701; ERR6054703-ERR6054706
Hi-C Illumina	ERR6054702
PolyA RNA-Seq Illumina	ERR9434973
*Genome assembly*	
Assembly accession	GCA_907165275.2
*Accession of alternate haplotype*	GCA_907165265.2
Span (Mb)	365.9
Number of contigs	68
Contig N50 length (Mb)	12.6
Number of scaffolds	55
Scaffold N50 length (Mb)	12.6
Longest scaffold (Mb)	16.1
BUSCO * genome score	C:97.8%[S:97.3%,D:0.5%],F:0.5%,M:1.7%,n:5286
*Genome annotation*	
Number of protein-coding genes	12,493
Average length of coding sequence (bp)	11,524.94
Average number of exons per transcript	6.70

*BUSCO scores based on the lepidoptera_odb10 BUSCO set using v5.2.2. C=complete [S=single copy, D=duplicated], F=fragmented, M=missing, n=number of orthologues in comparison. A full set of BUSCO scores is available at https://blobtoolkit.genomehubs.org/view/ilZygFili1.2/dataset/CAJRBF02/busco#Filters.

**Figure 3.**
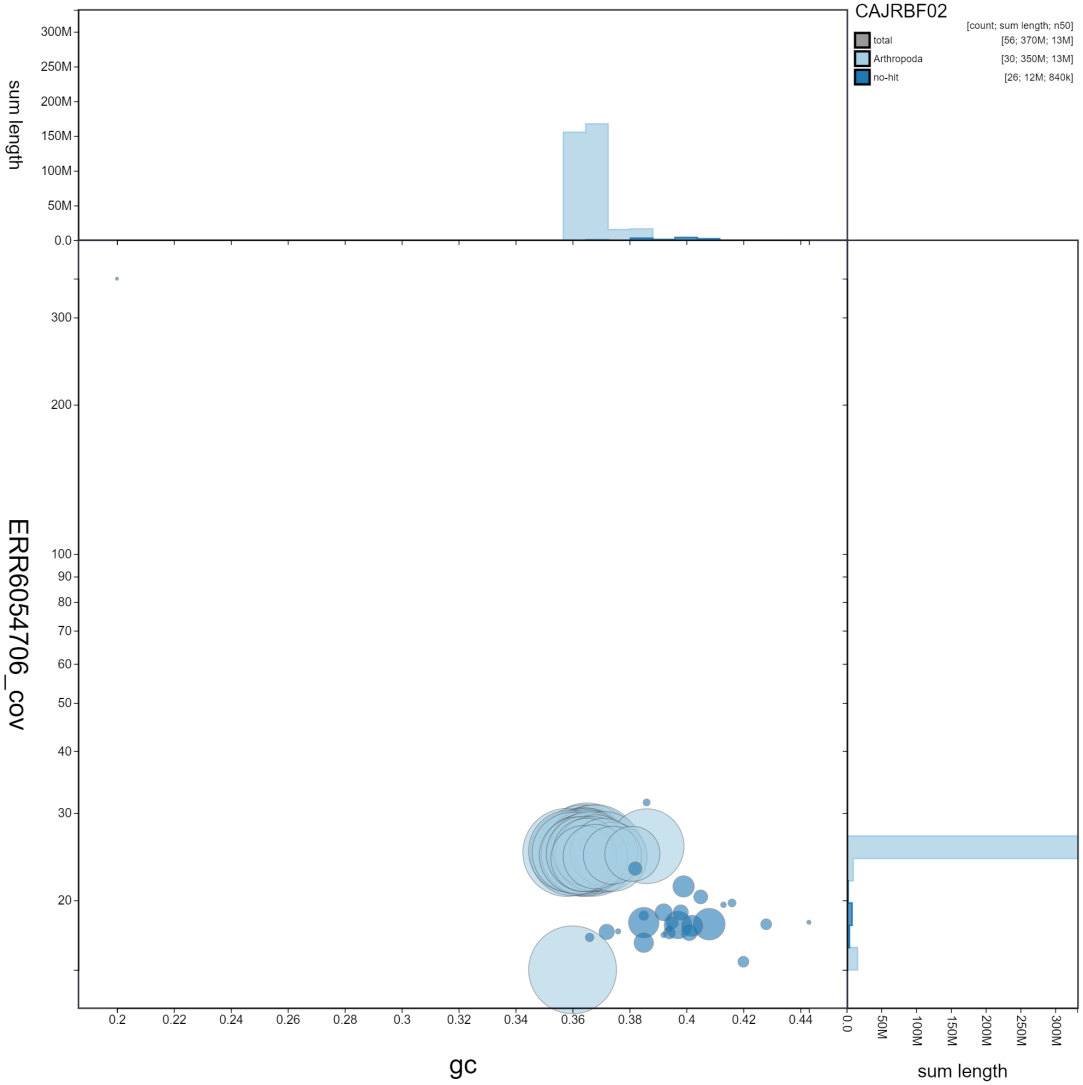
Genome assembly of *Zygaena filipendulae*, ilZygFili1.2: GC coverage. BlobToolKit GC-coverage plot. Scaffolds are coloured by phylum. Circles are sized in proportion to chromosome length Histograms show the distribution of chromosome length sum along each axis. An interactive version of this figure is available at https://blobtoolkit.genomehubs.org/view/ilZygFili1.2/dataset/CAJRBF02/blob#Filters.

**Figure 4.**
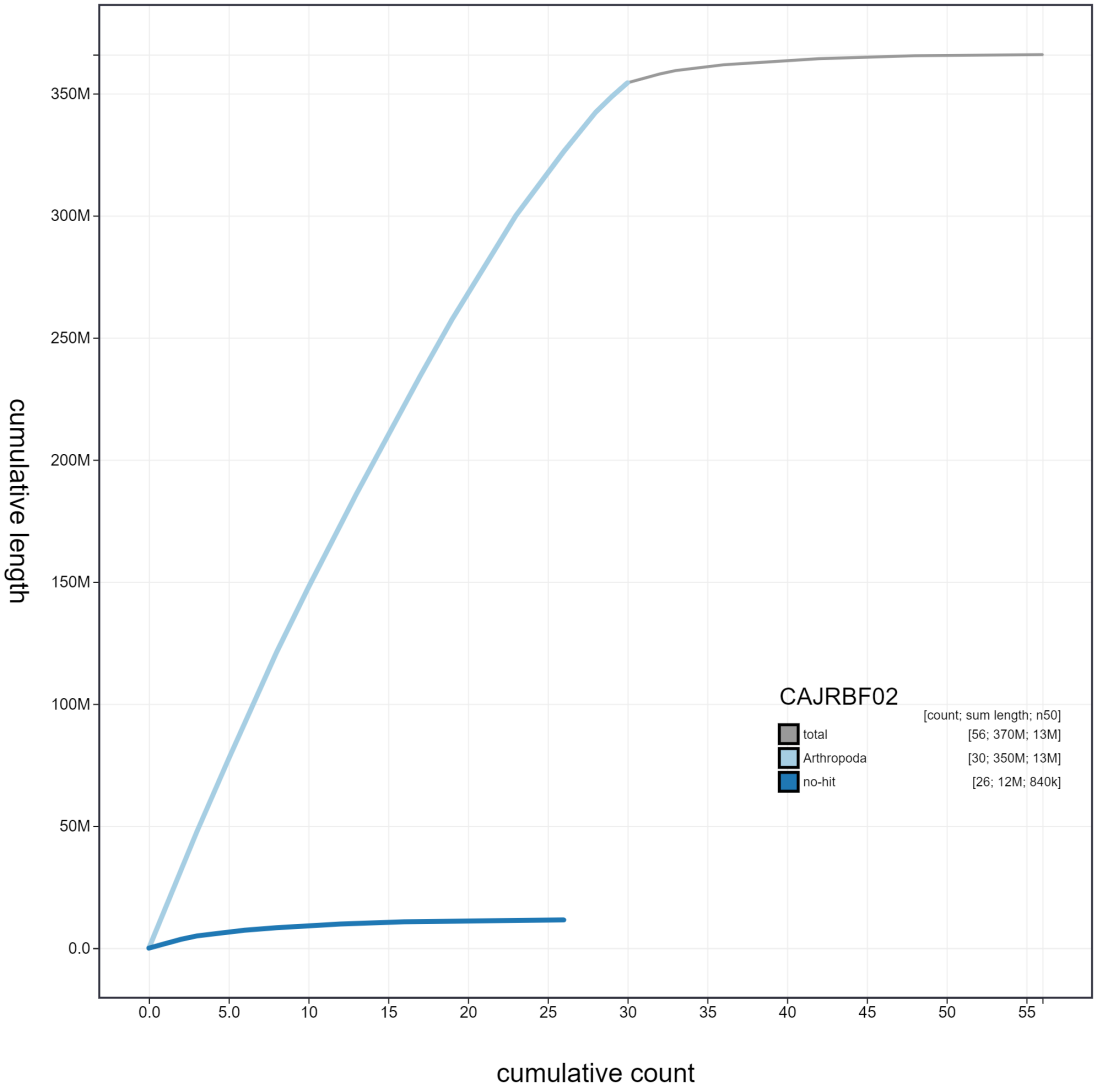
Genome assembly of *Zygaena filipendulae*, ilZygFili1.2: cumulative sequence. BlobToolKit cumulative sequence plot. The grey line shows cumulative length for all scaffolds. Coloured lines show cumulative lengths of scaffolds assigned to each phylum using the buscogenes taxrule. An interactive version of this figure is available at https://blobtoolkit.genomehubs.org/view/ilZygFili1.2/dataset/CAJRBF02/cumulative#Filters.

**Figure 5.**
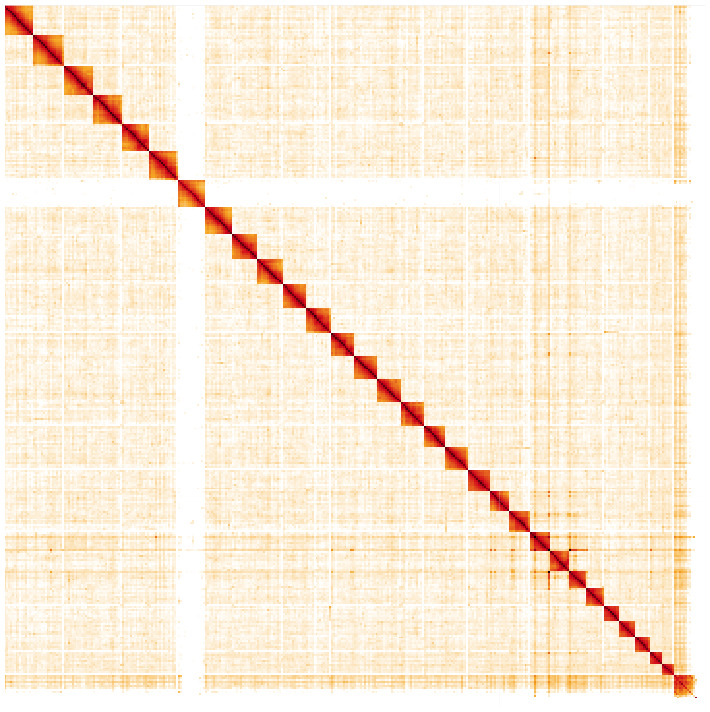
Genome assembly of *Zygaena filipendulae*, ilZygFili1.2: Hi-C contact map. Hi-C contact map of the ilZygFili1.2 assembly, visualised in HiGlass. Chromosomes are arranged in size order from left to right and top to bottom. The interactive Hi-C map can be viewed at https://genome-note-higlass.tol.sanger.ac.uk/l/?d=Aqyc_jJbQjuSzW9eMHqPQg.

**Table 2.** Chromosomal pseudomolecules in the genome assembly of *Zygaena filipendulae*, ilZygFili1.2.

INSDC accession	Chromosome	Size (Mb)	GC%
OU015649.1	1	16.1	36.5
OU015650.1	2	15.74	36.4
OU015651.1	3	15.74	36.7
OU015652.1	4	15.03	36.8
OU015653.1	5	14.95	36.4
OU015654.1	6	14.63	35.8
OU015656.1	7	14.34	36.3
OU015657.1	8	13.67	36
OU015658.1	9	12.99	36.5
OU015659.1	10	12.78	36.6
OU015660.1	11	12.77	35.9
OU015661.1	12	12.64	36.5
OU015662.1	13	12.32	36
OU015663.1	14	12.14	37.1
OU015664.1	15	11.96	36.3
OU015665.1	16	11.88	36.5
OU015666.1	17	11.8	36.6
OU015667.1	18	11.37	36.2
OU015668.1	19	10.74	36.8
OU015669.1	20	10.72	36.4
OU015670.1	21	10.57	38.6
OU015671.1	22	10.5	36.7
OU015672.1	23	9.02	37.4
OU015673.1	24	8.7	36.5
OU015674.1	25	8.53	37.1
OU015675.1	26	8.25	36.4
OU015676.1	27	7.85	36.8
OU015677.1	28	6.36	37.4
OU015678.1	29	5.72	38.1
OU015679.1	W	1.87	40.8
OU015655.1	Z	14.6	36
OU015680.1	MT	0.02	19.9
-	Unplaced	9.65	39.4

## Genome annotation report

The ilZygFili1.1 genome has been annotated using the Ensembl rapid annotation pipeline ( Table 1; GCA_907165275.1 https://rapid.ensembl.org/Zygaena_filipendulae_GCA_907165275.1/Info/Index). The resulting annotation includes 20,201 transcribed mRNAs from 12,493 protein-coding and 1,770 non-coding genes.

## Methods

### Sample acquisition and nucleic acid extraction

Two *Z. filipendulae* specimens (ilZygFili1, genome assembly; and ilZygFili3, RNA-Seq) were collected using a net from Ant Hills region and Wytham woods, Wytham, Berkshire, UK (latitude 51.765, longitude -1.327) by Douglas Boyes (University of Oxford). The specimens were identified by Douglas Boyes and snap-frozen on dry ice. A further *Z. filipendulae* specimen (ilZygFili2, Hi-C) was collected using a net from Wytham woods, Berkshire, UK (latitude 51.771, longitude -1.338) by Liam Crowley (University of Oxford). The specimen was identified by Liam Crowley and snap-frozen on dry ice.

DNA was extracted at the Tree of Life laboratory, Wellcome Sanger Institute. The ilZygFili1 sample was weighed and dissected on dry ice. Whole organism tissue was cryogenically disrupted to a fine powder using a Covaris cryoPREP Automated Dry Pulveriser, receiving multiple impacts. Fragment size analysis of 0.01–0.5 ng of DNA was then performed using an Agilent FemtoPulse. High molecular weight (HMW) DNA was extracted using the Qiagen MagAttract HMW DNA extraction kit. Low molecular weight DNA was removed from a 200-ng aliquot of extracted DNA using 0.8X AMpure XP purification kit prior to 10X Chromium sequencing; a minimum of 50 ng DNA was submitted for 10X sequencing. HMW DNA was sheared into an average fragment size between 12–20 kb in a Megaruptor 3 system with speed setting 30. Sheared DNA was purified by solid-phase reversible immobilisation using AMPure PB beads with a 1.8X ratio of beads to sample to remove the shorter fragments and concentrate the DNA sample. The concentration of the sheared and purified DNA was assessed using a Nanodrop spectrophotometer and Qubit Fluorometer and Qubit dsDNA High Sensitivity Assay kit. Fragment size distribution was evaluated by running the sample on the FemtoPulse system.

RNA was extracted from other abdomen tissue of ilZygFili3 in the Tree of Life Laboratory at the WSI using TRIzol, according to the manufacturer’s instructions. RNA was then eluted in 50 μl RNAse-free water and its concentration RNA assessed using a Nanodrop spectrophotometer and Qubit Fluorometer using the Qubit RNA Broad-Range (BR) Assay kit. Analysis of the integrity of the RNA was done using Agilent RNA 6000 Pico Kit and Eukaryotic Total RNA assay.

### Sequencing

Pacific Biosciences HiFi circular consensus and 10X Genomics Chromium read cloud sequencing libraries were constructed according to the manufacturers’ instructions. Sequencing was performed by the Scientific Operations core at the Wellcome Sanger Institute on Pacific Biosciences SEQUEL II (HiFi), Illumina NovaSeq 6000 (10X) and Illumina HiSeq 4000 (RNA-Seq) instruments. Hi-C data were generated in the Tree of Life laboratory from head/thorax tissue of ilZygFili2 using the Arima v2 kit and sequenced on a NovaSeq 6000 instrument.

### Genome assembly

Assembly was carried out with Hifiasm ( Cheng *et al.*, 2021); haplotypic duplication was identified and removed with purge_dups ( Guan *et al.*, 2020). One round of polishing was performed by aligning 10X Genomics read data to the assembly with longranger align, calling variants with freebayes ( Garrison & Marth, 2012). The assembly was then scaffolded with Hi-C data ( Rao *et al.*, 2014) using SALSA2 ( Ghurye *et al.*, 2019). The assembly was checked for contamination and corrected using the gEVAL system ( Chow *et al.*, 2016) as described previously ( Howe *et al.*, 2021). Manual curation ( Howe *et al.*, 2021) was performed using gEVAL, HiGlass ( Kerpedjiev *et al.*, 2018) and Pretext. The mitochondrial genome was assembled using MitoHiFi ( Uliano-Silva *et al.*, 2021), which performs annotation using MitoFinder ( Allio *et al.*, 2020). The genome was analysed and BUSCO scores generated within the BlobToolKit environment ( Challis *et al.*, 2020). Table 3 contains a list of all software tool versions used, where appropriate.

**Table 3.** Software tools used.

Software tool	Version	Source
Hifiasm	0.14-r312	Cheng *et al.*, 2021
purge_dups	1.2.3	Guan *et al.*, 2020
SALSA2	2.2	Ghurye *et al.*, 2019
longranger align	2.2.2	https://support.10xgenomics.com/ genome-exome/software/pipelines /latest/advanced/other-pipelines
freebayes	1.3.1-17- gaa2ace8	Garrison & Marth, 2012
MitoHiFi	2.11	Uliano-Silva *et al.*, 2021
HiGlass	1.11.6	Kerpedjiev *et al.*, 2018
PretextView	0.2.x	https://github.com/wtsi-hpag/ PretextView
BlobToolKit	3.0.5	Challis *et al.*, 2020

### Genome annotation

The Ensembl gene annotation system ( Aken *et al.*, 2016) was used to generate annotation for the *Z. filipendulae* assembly (GCA_907165275.1). Annotation was created primarily through alignment of transcriptomic data to the genome, with gap filling via protein-to-genome alignments of a select set of proteins from UniProt ( UniProt Consortium, 2019).

### Ethics/compliance issues

The materials that have contributed to this genome note have been supplied by a Darwin Tree of Life Partner. The submission of materials by a Darwin Tree of Life Partner is subject to the Darwin Tree of Life Project Sampling Code of Practice. By agreeing with and signing up to the Sampling Code of Practice, the Darwin Tree of Life Partner agrees they will meet the legal and ethical requirements and standards set out within this document in respect of all samples acquired for, and supplied to, the Darwin Tree of Life Project. Each transfer of samples is further undertaken according to a Research Collaboration Agreement or Material Transfer Agreement entered into by the Darwin Tree of Life Partner, Genome Research Limited (operating as the Wellcome Sanger Institute), and in some circumstances other Darwin Tree of Life collaborators.

## Data availability

European Nucleotide Archive: Zygaena filipendulae (6-spot burnet). Accession number PRJEB44832; https://identifiers.org/ena.embl/PRJEB44832.

The genome sequence is released openly for reuse. The *Z. filipendulae* genome sequencing initiative is part of the Darwin Tree of Life (DToL) project. All raw sequence data and the assembly have been deposited in INSDC databases. Raw data and assembly accession identifiers are reported in Table 1.

## Author information

Members of the University of Oxford and Wytham Woods Genome Acquisition Lab are listed here: https://doi.org/10.5281/zenodo.6418202.

Members of the Darwin Tree of Life Barcoding collective are listed here: https://doi.org/10.5281/zenodo.6418156.

Members of the Wellcome Sanger Institute Tree of Life programme are listed here: https://doi.org/10.5281/zenodo.6418327.

Members of Wellcome Sanger Institute Scientific Operations: DNA Pipelines collective are listed here: https://doi.org/10.5281/zenodo.5746904.

Members of the Tree of Life Core Informatics collective are listed here: https://doi.org/10.5281/zenodo.6125046.

Members of the Darwin Tree of Life Consortium are listed here: https://doi.org/10.5281/zenodo.6418363.
