# Supplementary material for: The genome sequence of the 6-spot burnet, Zygaena filipendulae (Linnaeus, 1758)
Source: Wellcome Open Res. 2024 Mar 18;7:197. Originally published 2022 Aug 1. [Version 2] doi: 10.12688/wellcomeopenres.17924.2 (PMC11422757; doi:10.12688/wellcomeopenres.17924.2)
Supplement: Peer review history

# Reviewer report: Reviewer response for version 2

**DOI:** 10.21956/wellcomeopenres.23185.r115617

**Published:** 2025-02-01

**Authors:** Bin Zhang

**Affiliations:** 1 China-Australia Joint Institute of Agricultural and Environmental Health, Qingdao Agricultural University, Shenzhen, China

This manuscript presents a high-quality, chromosome-level genome assembly of Zygaena filipendulae (6-spot burnet moth), a species of ecological and evolutionary significance due to its cyanogenic glucoside biosynthesis capabilities. The assembly spans 365.9 Mb, scaffolds into 31 chromosomal pseudomolecules (including W/Z sex chromosomes), and includes a complete mitochondrial genome (15.6 kb). The annotation identifies 12,493 protein-coding genes. The work leverages PacBio HiFi, 10X Genomics, Hi-C, and RNA-seq data, with transparent reporting of methods and data accessibility (ENA: PRIEB44832). Overall, the manuscript provides a valuable genomic resource for Lepidoptera research, chemical ecology, and conservation biology. There are still some points need to consider to improve as follows:

 Major Concerns and Recommendations

 1. Clarify why only abdominal RNA-seq data (ilZygFill3) was used for annotation. Were thorax/head tissues considered?

 2. Address whether public Zygaena transcriptomes (e.g., ENA datasets) were incorporated to improve annotation.

 3. Specify DNA extraction methods for the Hi-C sample (ilZygFill2), currently omitted in the "Sample acquisition" section.

 4. Expand the "Background" to emphasize Z. filipendulae’s conservation status (e.g., population declines, IUCN Red List) and the role of cyanogenic glucosides in mate selection, larval defense, and nitrogen metabolism (cite Zagrobelny et al., 2007, 2014; Bergman et al., 2020).

 5. Gene Annotation Gaps: Highlight unresolved questions (e.g., cyanogenic glucoside transporters, detoxification genes) that this genome could address.

 6. Figure 1: Remove the tube image (label mismatch) and retain only the specimen photo. Specify which specimen (ilZygFill1/2/3) is shown.

 7. Table 1: Include metadata for non-coding genes (1,770) and mitochondrial genome length in the main text.

 8. Update the MitoHiFi citation to the published version (Uliano-Silva et al., 2023, BMC Bioinformatics).

 9. Add citations for morphological descriptions (e.g., "six red spots") and cyanogenic enzyme studies (Zagrobelny et al., 2019).

 10. Revise "50 μl RNAse-free water" → "50 μl of RNase-free water" and "its concentration RNA assessed" → "its concentration was assessed."

 11. Correct "ilZygFill1.1 genome" to "ilZygFill1.2" in the "Genome annotation report."

 12. Metadata in ENA: Ensure RNA-seq sample metadata (e.g., sex, tissue type) are explicitly stated in ENA records.

 13. Keywords: Add "Lepidoptera" or "moth" to improve searchability.

 14. Ethics Statement: Clarify compliance with local/national guidelines for specimen collection.

 This manuscript provides a high-quality genome resource for Zygaena filipendulae, with significant potential to advance research in chemical ecology, Lepidoptera genomics, and conservation. Addressing the above concerns—particularly annotation validation, methodological clarity, and ecological context—will strengthen the manuscript’s impact and reproducibility.

Are sufficient details of methods and materials provided to allow replication by others?

Yes

Is the rationale for creating the dataset(s) clearly described?

Yes

Are the datasets clearly presented in a useable and accessible format?

Yes

Are the protocols appropriate and is the work technically sound?

Yes

Reviewer Expertise:

entomology, bioinformatics

I confirm that I have read this submission and believe that I have an appropriate level of expertise to confirm that it is of an acceptable scientific standard.

# Reviewer report: Reviewer response for version 2

**DOI:** 10.21956/wellcomeopenres.23185.r114878

**Published:** 2024-12-19

**Authors:** Shiqi Luo

**Affiliations:** 1 Department of Entomology, College of Plant Protection, China Agricultural University, Beijing, China

This study presents the chromosomal-level genomic information of the six-spot burnet moth *Zygaena filipendulae*. The sequencing data includes Pacific Biosciences single-molecule long reads, 10X Genomics reads, Hi-C and RNA-seq data. The assembly quality is high. Table 1 provides essential information and data storage details, and Table 3 specifies the software used.

 I have a few comments

 1) In the sentence “The ilZygFili1.1 genome has been annotated using the Ensembl rapid annotation pipeline” in the "Genome annotation report" part，should the name "ilZygFili1.1" be revised to "ilZygFili1.2" ?

 2) In the sentence“RNA was then eluted in 50 μl RNAse-free water and its concentration RNA assessed using a Nanodrop spectrophotometer and Qubit Fluorometer using the Qubit RNA Broad-Range (BR) Assay kit.”, the correct grammar should be “50 μl of RNAse-free water” and “its concentration was assessed”

 3) In the “Genome annotation” part, “with gap filling via protein-to-genome alignments of a select set of proteins from UniProt (UniProt Consortium, 2019).”, What is “a select set of protein from Uniprot”? Please specify.

 4) Please provide more specific parameter information for the software listed in Table 3.

 5) The Reference “Uliano-Silva M, Nunes JGF, Krasheninnikova K, et al.: marcelauliano/MitoHiFi:  mitohifi_v2.0. 2021.” should be replaced with the published one: Uliano-Silva, M., Ferreira, J.G.R.N., Krasheninnikova, K. *et al.* MitoHiFi: a python pipeline for mitochondrial genome assembly from PacBio high fidelity reads. *BMC Bioinformatics* **24**, 288 (2023). https://doi.org/10.1186/s12859-023-05385-y

Are sufficient details of methods and materials provided to allow replication by others?

Partly

Is the rationale for creating the dataset(s) clearly described?

Yes

Are the datasets clearly presented in a useable and accessible format?

Yes

Are the protocols appropriate and is the work technically sound?

Yes

Reviewer Expertise:

functional genomics, non-coding RNAs, gut microbiome

I confirm that I have read this submission and believe that I have an appropriate level of expertise to confirm that it is of an acceptable scientific standard.

# Reviewer report: Reviewer response for version 2

**DOI:** 10.21956/wellcomeopenres.23185.r97889

**Published:** 2024-09-24

**Authors:** Sivasankaran Kuppusamy

**Affiliations:** 1 Loyola College, Chennai, Tamil Nadu, India

Authors have sequenced the whole genome of the 6-spot burnet *, Zygaena filipenudlae* (Linnaeus, 1758). Totally 365.9 megabases received through the assembly.  31 chromosomes, 15.6 kilobases of mitochondrial genome sequences, 12493 protein-coding sequences, 20,201 transcribed mRNAs and 1,770 non-coding genes were received through the genome assembly and annotations. Authors have used the appropriate software for the genome assembly and annotation.

**Comments on the manuscript**

- Authors haven’t been given the transcribed mRNAs and non-coding genes in Table no 1. 
* *
- Total mitochondrial genome sequences haven't been given in the text. 
- Above all, I confirm that the manuscript meets the necessary scientific standard and is suitable for indexing"

Are sufficient details of methods and materials provided to allow replication by others?

Yes

Is the rationale for creating the dataset(s) clearly described?

Yes

Are the datasets clearly presented in a useable and accessible format?

Yes

Are the protocols appropriate and is the work technically sound?

Yes

Reviewer Expertise:

Phylogenetic analysis of Lepidopteran Noctuoidea moths using mitochondrial genome sequence

I confirm that I have read this submission and believe that I have an appropriate level of expertise to confirm that it is of an acceptable scientific standard.

# Reviewer report: Reviewer response for version 1

**DOI:** 10.21956/wellcomeopenres.19862.r56023

**Published:** 2023-10-06

**Authors:** Érika de Castro

**Affiliations:** 1 Zoology, Cambridge University, Cambridge, Cambridgeshire, UK

This article is a report of genome sequencing and assembly of *Zygaena filipendulae* (Assembly identifier: ilZygFili1.2). Three specimens were used in this study, each for a different type of sequencing: ilZygFili1 for Hi-Fi, ilZygFili2 for Hi-C and ilZygFili3 for RNA-seq. HiFi produces highly accurate long reads which are ideal for a good quality assembly and it was combined with Hi-C to improve assembly at chromosomic level. The RNA-seq data generated in this study was used to make a gene annotation for the new assembly. All software/packages used in this project are listed in the methods and all the generated data is available on ENA (PRJEB44832). The assembly has 31 pseudo chromosomes which is the chromosome number of many related moths. As a female was used for the genome assembly, its sex chromosomes were heterozygous and the W and Z chromosome were also assembled in this genome. This genome assembly will be very important not only for lepidopterists, but also conservation biologists and chemical ecologists: *Z. filipendulae* is one of the most representative species of the *Zygaena* genus and these moths are in decline, with some species already in the red list. Moreover, this species has become a model to study the evolution of cyanogenesis in insects. I think this two topics could be better explained in the rationale for creating the dataset. I, hereby, recommend the article, but do encourage the authors to improve the “Background“ session and also have some minor questions and suggestions:

**Format**

 Line numbers in the pdf would facilitated for reviewers to comment on specific sentences.

**Keywords**

 You could add 'moth' or 'Lepidoptera'

**Background**

- In “ *Some Zygaena species have become model organisms to study the evolution of chemical defence compounds*”, the word “compound” is pleonasm, I would say ‘chemical defences’ instead.
- Missing reference for the sentence " *Forewings of Z. filipendulae are black and distinctively marked with six red spots.*”
- Please rephrase as ‘ …are two cytochrome P450s and a UDP-glycosyltransferase

Why is it important to “understand the evolution of cyanogenic glucoside biosynthesis”? As a chemical ecologist, I know this answer and I am very interested on this subject, but I think this would not be appealing for a broad audience. I would rephrase this session focusing on: (1) Populations of Zygaena moths are in decline, with some species even in the red list (Bergman et al., 2020), (2) Cyanogenic glucosides played an important role not only in the diversification Zygaena moths (aposematism and mimicry), but also in its life-history (Females prefer to mate with the most toxic males (Zagrobelny, Bak, Olsen, et al., 2007); They use these compounds as nuptial gifts which are then transferred to their eggs to protect their offspring (Zagrobelny et al., 2014); Larvae balance between biosynthesis and sequestration of cyanogenic glucosides depending on how much of these compounds they can get from their hostplant, but biosynthesis has higher fitness cost (Zagrobelny, Bak, Thorn Ekstrøm, et al., 2007)), and (3) There is a lack of knowledge on the genetic basis of their cyanogenic metabolism and mechanism that allows these compounds to play multiple roles in the biology of *Zygaena filipendulae*. In my opinion, it is not only about how they evolve to de novo biosynthesize their cyanogenic glucosides (CGs), but to sequester CGs (unknown transporter), to balance between biosynthesis and sequestration (unknown), to activate CGs to release cyanide (B-glu), to detoxify cyanide (B-Cas and Rhodanase), to turn-over the CGs when they need nitrogen (unknown), to perceive the CGs in their hostplant and their mate-partners (unknown), etc. The *Z. filipendulae* genome can aid studies in this broad aspect of the evolution of cyanogenic metabolism.

**Methods**

Sample acquisition and nucleic acid extraction:

- You don’t need to specify which author collected and identified which samples.
- Add the manufacturer of TRIzol.
- Currently, there is no explanation in this session about the method used for the DNA extraction of ilZygFili2 for Hi-C. Please add that.
- I checked ENA and I could not find the information on sex and tissue of the sample used for transcriptomics. Please added this metadata info in ENA, if this is not there already, so the data to facilitated reuse.

Questions: Only the abdomen of the specimen ilZygFili3 were used for the RNA extraction and analyses and I would like to know the rationale for this decision. Moreover, there are other transcriptomic datasets of *Zygaena filipendulae* that are available at ENA and these could have being used to improve even more the genome annotation – why were these not used for the annotation?

**Figures**

 Figure 1. Please keep just the first photo of moth where it is possible to see the six red dots and remove the second one and the tube photo. The tube photo could go to supplementary material, if there is tissue left and you think this info could be used in case other researchers want to re-sequence the same sample – otherwise it is irrelevant for the content of the paper and even confusing, as the tube has a label (FF03455026) that it is different from the samples code (ilZygFili). The legend also does not say to which specimen the photo correspond to (ilZygFili 1, 2 or 3?)

**References:**

- Bergman, K. O., Burman, J., Jonason, D., Larsson, M. C., Ryrholm, N., Westerberg, L., & Milberg, P. (2020). Clear-cuts are temporary habitats, not matrix, for endangered grassland burnet moths (Zygaena spp.). *Journal of Insect Conservation*, *24*(2), 269–277. https://doi.org/10.1007/S10841-019-00193-3/FIGURES/2
- Zagrobelny, M., Bak, S., Olsen, C. E., & Møller, B. L. (2007). Intimate roles for cyanogenic glucosides in the life cycle of Zygaena filipendulae (Lepidoptera, Zygaenidae). *Insect Biochemistry and Molecular Biology*, *37*(11), 1189–1197. https://doi.org/10.1016/J.IBMB.2007.07.008
- Zagrobelny, M., Bak, S., Thorn Ekstrøm, C., Erik Olsen, C., & Lindberg Møller, B. (2007). The cyanogenic glucoside composition of Zygaena filipendulae (Lepidoptera: Zygaenidae) as effected by feeding on wild-type and transgenic lotus populations with variable cyanogenic glucoside profiles. *Insect Biochemistry and Molecular Biology*, *37*(1), 10–18. https://doi.org/10.1016/J.IBMB.2006.09.008
- Zagrobelny, M., Olsen, C. E., Pentzold, S., Fürstenberg-Hägg, J., Jørgensen, K., Bak, S., Møller, B. L., & Motawia, M. S. (2014). Sequestration, tissue distribution and developmental transmission of cyanogenic glucosides in a specialist insect herbivore. *Insect Biochemistry and Molecular Biology*, *44*(1), 44–53. https://doi.org/10.1016/J.IBMB.2013.11.003

Are sufficient details of methods and materials provided to allow replication by others?

Yes

Is the rationale for creating the dataset(s) clearly described?

Partly

Are the datasets clearly presented in a useable and accessible format?

Yes

Are the protocols appropriate and is the work technically sound?

Yes

Reviewer Expertise:

Chemical ecologist

I confirm that I have read this submission and believe that I have an appropriate level of expertise to confirm that it is of an acceptable scientific standard, however I have significant reservations, as outlined above.

## References

[1] : Clear-cuts are temporary habitats, not matrix, for endangered grassland burnet moths (Zygaena spp.). *Journal of Insect Conservation* .2020;24(2) : 10.1007/s10841-019-00193-3 269-277 10.1007/s10841-019-00193-3

[2] : Intimate roles for cyanogenic glucosides in the life cycle of Zygaena filipendulae (Lepidoptera, Zygaenidae). *Insect Biochem Mol Biol* .2007;37(11) : 10.1016/j.ibmb.2007.07.008 1189-97 10.1016/j.ibmb.2007.07.008 17916505

[3] : The cyanogenic glucoside composition of Zygaena filipendulae (Lepidoptera: Zygaenidae) as effected by feeding on wild-type and transgenic lotus populations with variable cyanogenic glucoside profiles. *Insect Biochem Mol Biol* .2007;37(1) : 10.1016/j.ibmb.2006.09.008 10-8 10.1016/j.ibmb.2006.09.008 17175442

[4] : Sequestration, tissue distribution and developmental transmission of cyanogenic glucosides in a specialist insect herbivore. *Insect Biochem Mol Biol* .2014;44: 10.1016/j.ibmb.2013.11.003 44-53 10.1016/j.ibmb.2013.11.003 24269868

# Reviewer report: Reviewer response for version 1

**DOI:** 10.21956/wellcomeopenres.19862.r66242

**Published:** 2023-10-05

**Authors:** Rachel Steward

**Affiliations:** 1 Biology, Lunds Universitet, Lund, Skåne County, Sweden

Boyes & Crowley present a chromosome level, highly complete genome and  preliminary gene annotation for *Zygaena filipendulae*, well-studied aposematic moth. This is the only available reference genome for the genus, and represents an important resource for understanding the genomic basis of  cyanogenic glucosides sequestration and biosynthesis. Assembly, annotation and data are available from ENA and Darwin Tree of Life, and this genome note provides adequate information about sequencing and assembly methods and final assembly quality. I especially appreciated the table of tools and versions used, with citations. I have several minor comments, but overall this is a useful and concise contribution.

 Minor comments:

1. The annotation deserves some kind of quality assessment, e.g., BUSCO on the protein coding transcripts. The authors also failed to mention that the sex of the sample for RNAseq was unknown, and thus it is unclear whether annotation of the W chromosome was also informed by gene expression data.
2. More detail on the gene annotation methods would be useful, including RNA source tissue (thorax, available on ToLQC) and RNA library prep, a brief synopsis of the Ensembl gene annotation system (which aligner was used, which gene prediction software, which protein databases, etc.), whether this annotation includes UTRs, etc.
3. The number of Lepidoptera_odb10 SCOs is 5286 (Manni et al. 2021. Mol Biol Evol). Boyes & Crowley cite it as 954. Either the information on which database was used is incorrect, or the number of SCOs used is incorrect. Please revise.

Are sufficient details of methods and materials provided to allow replication by others?

Partly

Is the rationale for creating the dataset(s) clearly described?

Yes

Are the datasets clearly presented in a useable and accessible format?

Yes

Are the protocols appropriate and is the work technically sound?

Yes

Reviewer Expertise:

Gene expression, alternative splicing, plant-insect interactions, population genetics, ecological genomics

I confirm that I have read this submission and believe that I have an appropriate level of expertise to confirm that it is of an acceptable scientific standard, however I have significant reservations, as outlined above.

# Reviewer report: Reviewer response for version 1

**DOI:** 10.21956/wellcomeopenres.19862.r56020

**Published:** 2023-05-03

**Authors:** Wannes Dermauw

**Affiliations:** 1 Plant Sciences, Flanders Research Institute for Agriculture, Fisheries and Food, Merelbeke, Oost-Vlaanderen, Belgium

Boyes et al. report the genome sequence of a moth species belonging to the Zygaenidae, a family that has been well studied for the evolution of chemical defense compounds. The genome was sequenced from a single female, with a genome assembly size of 365.9 MB. RNAseq and Hi-C data were generated from other specimens and used for annotation and chromosome conformation, respectively. This is a clear and concise report that merits indexing as a data note. I only have a few minor comments:

- Authors provided a BUSCO genome score, but ideally also calculate a BUSCO score for the predicted gene set (12,493 genes). A brief comparison with other sequenced lepidopteran genomes would also be recommended.
- Page 4: it is not clear to me how a reader can obtain access to the predicted gene set; authors should upload a gff file and the predicted CDS to NCBI or include this data as a supplementary file(s)
- Page 3, 1st paragraph: add the total number of reads that were generated using each sequencing technology
- Page 3: authors should briefly explain how they determined W and Z sex chromosomes
- Methods/Genome Assembly: This section is very general, and I would suggest the authors to include scripts or more details re software settings that were used.

Are sufficient details of methods and materials provided to allow replication by others?

Partly

Is the rationale for creating the dataset(s) clearly described?

Yes

Are the datasets clearly presented in a useable and accessible format?

Yes

Are the protocols appropriate and is the work technically sound?

Yes

Reviewer Expertise:

molecular entomology, genomics,  horizontal gene transfer, pesticide resistance

I confirm that I have read this submission and believe that I have an appropriate level of expertise to confirm that it is of an acceptable scientific standard, however I have significant reservations, as outlined above.
